# Investigation of the efficacy and safety of cryoablation and intra-arterial PD-1 inhibitor in patients with advanced disease not responding to checkpoint inhibitors: An exploratory study

**DOI:** 10.3389/fimmu.2022.990224

**Published:** 2022-09-23

**Authors:** Fuqun Wei, Rui Guo, Yuan Yan, Ruixiang Lin, Jin Chen, Zhengyu Lin

**Affiliations:** ^1^The Department of Interventional Radiology, First Affiliated Hospital of Fujian Medical University, Fuzhou, Fujian, China; ^2^Fujian Provincial Key Laboratory of Precision Medicine for Cancer, Fuzhou, Fujian, China

**Keywords:** solid cancer, cryoablation, immune resistance, arterial perfusion, PD-1

## Abstract

**Objective:**

To explore the effectiveness of cryoablation combined with arterial perfusion with programmed cell death protein 1 inhibitors in overcoming immune resistance in advanced solid cancers.

**Methods:**

In this pilot retrospective study, nine patients with solid cancers were treated with tumour cryoablation and arterial perfusion with programmed cell death protein 1 inhibitors, which had previously proven ineffective. The CIBERSORT software was used to estimate the levels of tumour-infiltrating immune cells in the challenged tumour. Changes in the levels of circulating T cells were assessed using flow cytometry. The primary endpoints were disease control and objective response rates, and the secondary endpoint was safety.

**Results:**

The nine patients with advanced solid tumours received cryoablation combined with arterial perfusion with programmed cell death protein 1 inhibitors between June and December 2021. The median follow-up time was 5.8 months. We recorded an objective response rate in two patients (22.22%). The best overall responses were partial responses in two patients (22.22%) and one case (11.11%) of stable disease, while six patients (66.67%) presented progressive disease. However, the median overall survival time was not reached. The median progression-free survival was 2.4 months. Treatment-related severe adverse events included one case of abdominal infection and one case of upper gastrointestinal bleeding, which were cured after the intervention. The CIBERSORT software confirmed the importance of cryoablation in regulating tumour-infiltrating immune cells. Thus, macrophage polarisation from the M2 to the M1 phenotype in the challenged tumour and a gradual increase in the levels of circulating CD4^+^ T cells were observed after administration of the combination therapy.

**Conclusion:**

Cryoablation combined with arterial perfusion with programmed cell death protein 1 inhibitors has the potential efficacy and safety to overcome immune resistance in patients with advanced solid cancers. The combination therapy leads to macrophage polarisation from the M2 to the M1 phenotype in the challenged tumour to enhance antitumour immunity.

## Introduction

Immune checkpoint inhibitors (ICIs) have revolutionised the field of cancer treatment, and a variety of ICI-based therapies, such as combination chemotherapy and combination local therapy, have brought new strategies to treat solid tumours. While classical chemotherapy and radiotherapy remain the mainstay of treatment for most solid cancers, several well-designed clinical trials have shown the potential of ICIs as a first-line treatment for a wide range of solid tumours (1–5). ICIs activate the immune system and generate antitumour immune responses predominantly by releasing suppressive brakes of T cells. However, studies have shown that although T cells are a cornerstone of ICI therapy, they also activate other innate and adaptive immune cells, all of which are involved in and coordinate the control of tumours, as well as possible tumour responses.

To date, various ICI agents, which target cytotoxic T-lymphocyte-associated protein 4, programmed cell death protein 1 (PD-1), and programmed death-ligand 1 (PD-L1), have been approved by the U.S. Food and Drug Administration to treat a variety of solid tumours, such as urothelial carcinoma, non-small cell lung cancer, hepatocellular carcinoma, and Merkel cell carcinoma. However, current evidence shows that the response rates to ICI therapy vary considerably across different cancers, with primary resistance to immunotherapy ranging from more than 80% of patients with refractory Hodgkin’s lymphoma to a much larger proportion of patients with colorectal cancer (6, 7). Furthermore, patients who initially respond to ICIs may experience tumour progression during subsequent treatment, known as acquired resistance (8). These complex resistance mechanisms limit the duration of ICI effectiveness; thus, it is critical to develop combination therapies that may allow more patients to benefit from immunotherapy after developing resistance.

Spontaneous regression of metastases from primary prostate tumours after cryoablation was reported in the 1970s, and the results suggested that this regression could be immune-mediated (9, 10). Cryoablation induces tumour cell death *via* osmotic shock or physical damage from ice crystals, resulting in the release of cellular contents into the extracellular space and in the activation of immune responses. Preclinical animal experiments have shown that cryoablation therapy significantly inhibits tumour recurrence and increases tumour sensitivity to ICIs (11–13). Cryoablation therapy has shown promise in the treatment of advanced breast, lung, liver, glioma, melanoma, and pancreatic cancers (14–17). However, there have been no definitive investigations to determine whether cryoablation can overcome immune resistance in solid tumours. This study aimed to investigate the safety and efficacy of a combined therapeutic strategy including cryoablation and arterial perfusion with PD-1 inhibitors in patients with solid tumours after immunotherapy failure.

## Materials and methods

### Patient selection

The medical records of nine consecutive patients whose advanced solid tumours had progressed and who received combined cryoablation and arterial perfusion therapy with PD-1 inhibitors at the First Affiliated Hospital of Fujian Medical University between June and December 2021 were reviewed. Treatment was determined based on the patient’s wishes and the advice of a multidisciplinary team consisting of oncologists, interventional radiologists, radiologists, and surgeons. The inclusion criteria were as follows: (a) a histological diagnosis of a malignant tumour; (b) unresectable or distant metastasis; (c) failure of previous PD-1 inhibitor therapy; (d) a score of 0 or 1 on the Eastern Cooperative Oncology Organization Performance Scale (ECOG-PS); (e) at least one measurable lesion as defined by the Response Evaluation Criteria in Solid Tumours (RECIST) version 1.1; (f) no active or documented history of autoimmune diseases; and (g) at least one course of treatment completed in the combination phase. The exclusion criteria were as follows: (a) additional treatment prior to disease progression and (b) an ECOG-PS score of ≥ 2. This study was conducted in accordance with the ethical guidelines of the 1975 Declaration of Helsinki and was approved by the Ethics Committee of the Hospital (No.: MRCTA, ECFAH of FMU (2022) 246).

### Intervention

Each patient received one cycle of combination therapy as follows: on day 1, one or two lesions were completely cryoablated or incompletely cryoablated if they were larger than 5 cm, and the remaining lesions were left untreated; on day 3 (within 48 h), local arterial perfusion with a PD-1 inhibitor was performed in the ablation target organ. Local arterial perfusion with PD-1 inhibitors was subsequently recommended every 3 weeks until the occurrence of disease progression, unacceptable toxicity, or failure to follow up. Patients who were evaluated for progression for the first time could be administered a second combination therapy, if desired. Previous combination treatments, including chemotherapy and tyrosine kinase inhibitors, remained unchanged.

Cryoablation was performed using a HYG KB-II system (HYGEA Medical, Hong Kong, China). The target lesions were selected by interventional radiologists with more than 10 years of experience in percutaneous cryoablation, based on technical factors, such as the distance from major intrahepatic vessels, proximity to adjacent organs, ease of needle insertion, and patient’s symptoms, such as pain. All surgeries were performed under local anaesthesia with 2% lidocaine. A cryoprobe was inserted into the target lesion under computerised tomography (CT) guidance. The liquid-nitrogen cryogenic system was then activated, and the temperature of the tip was rapidly reduced to between −180 and −196°C to form a iceball, which was frozen for 10–15 min. A CT scan was further performed intraoperatively to confirm that the iceball covered the tumour area. An anhydrous ethanol active thawing system was then turned on to reach a temperature of 50–80°C, and active thawing was carried out for 5–15 min. The cryoablation and active thawing cycles were repeated twice. After cryoablation, a CT scan was performed again to determine whether bleeding, pneumothorax, or other complications had occurred.

Transarterial infusion of PD-1 inhibitors was performed with a 2.8-F microcatheter (Boston Scientific Corporation, Natick, MA, USA), which was superselectively placed into the feeding artery of the organ corresponding to the ablated tumour, and the artery was uniformly infused with a PD-1 inhibitor and 100 mL of normal saline for approximately 2 h (50 mL/h).

### Patient follow-up

The antitumour response was evaluated according to the RECIST 1.1 guidelines (18). The first on-study radiographic examination (contrast enhanced CT or MRI, PET-CT if necessary) was conducted at week 8, and subsequent examinations were conducted every 3 weeks during the treatment course, until the occurrence of disease progression or treatment discontinuation. Complete or partial responses were further confirmed at least 4 weeks after the first response. Objective response was defined as the proportion of patients who achieved a complete response plus partial response as their best overall response, and disease control was defined as the proportion of patients who achieved a complete response plus partial response plus stable disease as their best overall response. Patients were monitored for overall survival every 30 days after disease progression or treatment discontinuation until the occurrence of death or loss to follow-up. Safety was assessed by vital signs, laboratory measurements, adverse events, serious adverse events, and adverse events of special interest. Adverse events were assessed according to the Common Terminology Criteria for Adverse Events (CTCAE), version 5.0 (19).

### Evaluation of systemic immune responses in peripheral blood

During treatment, it was recommended that the blood be collected one day before cryoablation and one day after each arterial perfusion. One patient (11.1%) received continuous blood transfusions during treatment. After serum starvation for 24 h, cells were divided into two groups. One was treated with H_2_O_2_ (400 M) for 12 h to induce cell apoptosis; afterwards, 0.5–1.9 × 10^6^ cells were collected by centrifugation and incubated with annexin V/propidium iodide using an Annexin V-FITC/PI cell apoptosis detection kit (TransGen Biotech, Beijing, China) according to the manufacturer’s instructions. After incubation for 15 min in the dark, the cells were analysed using flow cytometry. Early apoptotic cells were stained with annexin V alone, whereas necrotic and late apoptotic cells were stained with both annexin V and propidium iodide.

### Transcriptome analysis

Tumour biopsy specimens were obtained from one patient (11.1%) before and after cryoablation. Biopsy specimens of the burden tumour and matched peripheral blood were collected from the patient before the start of cryoablation and after three cycles of arterial perfusion, and RNA was extracted from the biopsy tissue according to the manufacturer’s instructions (TIANGEN, Beijing, China). The RNA library for sequencing was prepared as follows: mRNA was purified from total RNA using a poly(T) oligonucleotide and then fragmented into 300–350-bp fragments; the first cDNA strand was reverse-transcribed using the fragmented RNA as a template in the presence of dNTPs (dATP, dTTP, dCTP, and dGTP), and the synthesis of the second cDNA strand was subsequently performed. The remaining overhangs of the double-stranded cDNA were converted into blunt ends using exonuclease/polymerase activity. After adenylation of the 3′ ends of the DNA fragments, sequencing adaptors were ligated to the cDNA, and the library fragments were purified. The template was amplified by polymerase chain reaction, and the products were purified to obtain the final library. The libraries were sequenced using an Illumina NovaSeq 6000 platform (paired-end, 150 bp), and the sequencing data were subjected to standard data analysis. Finally, tumour-infiltrating immune cells were quantified using the CIBERSORT software.

### Statistical analysis

Overall survival was calculated from the commencement of cryotherapy to the date of the patient’s death or last follow-up. Progression-free survival was measured from the initiation of cryotherapy to the patient’s first confirmed radiological progression or death, whichever occurred first, or their last follow-up visit. Adverse events during treatment and follow-up were recorded and graded according to the CTCAE version 5.0 guidelines. Statistical analyses and graphing were performed using R version 3.6.1 (http://www.r-project.org/).

## Results

### Baseline characteristics of the patients

The baseline characteristics of the enrolled patients are shown in **Table 1**. The median age of the study population was 58 years (range: 38–75 years). Six patients had primary hepatocellular carcinoma, and the remaining three had adrenal leiomyosarcoma, gallbladder carcinoma, and pancreatic carcinoma, respectively. Eight patients (88.89%) had stage IV tumours with distant metastases. Four patients (44.44%) were subjected to complete and five (55.56%) to incomplete tumour ablation. Five patients (55.56%) had responded to prior immunotherapy, while four (44.44%) had no response.

**Table 1:** Baseline characteristics.

Characteristic	N = 9
Age, median	58 (38 – 75)
Gender (male/female)	8/1
ECOG-PS	
0	7
1	2
Tumor type	
Hepatocellular carcinoma	6
Gallbladder carcinoma	1
Pancreatic cancer	1
Adrenal carcinoma	1
TNM stage	
IIIA	1
IV	8
Distant metastases	
No	1
Yes	8
Cryoablation location of the tumor	
Liver	4
Lung	1
pelvic	1
Chest wall	2
Abdominal	1
Cryoablation method	
Complete	4
Incomplete	5
Type of immune resistant	
acquired resistance	5
primary resistance	4

ECOG-PS, Eastern Cooperative Oncology Group performance status.

### Efficacy and safety of combined cryoablation and arterial perfusion

**Figure 1:**
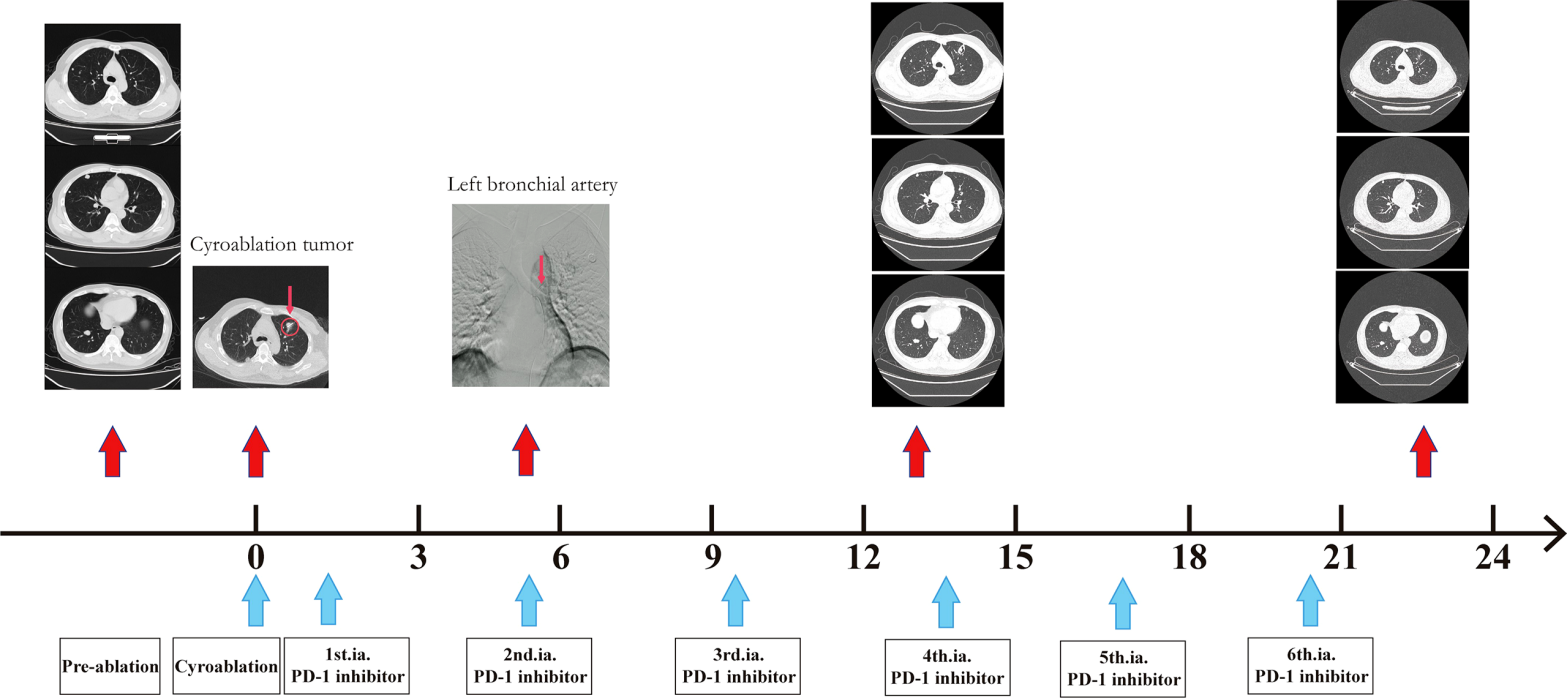
Example of partial patient response. A 45-year-old man with ruptured hepatocellular carcinoma underwent surgical resection. Lung metastasis occurred during the follow-up, and lung metastatic cancer increased and enlarged after more than one year of treatment with tyrosine kinase inhibitor drugs combined with PD-1 inhibitors. The patient agreed to undergo cryoablation (1 cycle) combined with arterial infusion of the PD-1 inhibitor (1 cycle/3 weeks). Before the fourth cycle of arterial perfusion therapy, a partial response emerged and has remained so far.

The best antitumour responses were a partial response in two patients, stable disease in one, and progressive disease in six (**Supplementary Table**). The objective response and disease control rates of the entire cohort were 22.2% and 33.3%, respectively (**Table 2**). The median progression-free survival time in the cohort was 3.17 (95% confidence interval, 1.25−5.09), and one patient has not progressed to date (**Figure 1**). The median overall survival was not reached, and the 6-month survival rate was 55.5%. The most common adverse event was pain associated with the tumour and with the ablation. Treatment-related severe adverse events included one case of abdominal infection and one case of upper gastrointestinal bleeding, which were cured after the intervention (**Table 3**). Rash and liver function impairment may be associated with ICI. Other side effects are mainly associated with ablation, the tumour itself, and other combined therapy.

**Table 2:** Best response to treatment according to RECIST 1.1.

Overall response, %	All treated participants (n = 9)
CR	0 (0%)
PR	2 (22.22%)
SD	1 (11.11%)
PD	6 (66.67%)
Objective Response Rate	22.22%
Disease Control Rate	33.33%
PFS (mean ± SD)	3.17 m (95%CI 1.25~5.09)
6-months OS, %	55.55%

RECIST, Response Evaluation Criteria in Solid Tumors; CR, complete response; PR, partial response; SD, stable disease; PD, progressive disease.

**Table 3:** Treatment-related adverse events.

Event	All grade	Grade1/2	Grade3/4
Weight loss	3 (33.3%)	3 (33.3%)	0(0%)
Decreased appetite	2 (22.22%)	2 (22.22%)	0(0%)
Nausea	1 (11.11%)	1 (11.11%)	0(0%)
Weakness	1 (11.11%)	1 (11.11%)	0(0%)
Rash	2 (22.22%)	2 (22.22%)	0(0%)
Infection	1 (11.11%)	0 (0%)	1(11.11%)
Hemorrhage	2 (22.22%)	1 (11.11%)	1(11.11%)
Pain	5 (50.50%)	5 (50.50%)	0(0%)
Hypothyroidism	0 (0%)	0 (0%)	0(0%)
Renal insufficiency	0 (0%)	0 (0%)	0(0%)
Abnormal hepatic function	2 (22.22%)	2 (22.22%)	0(0%)
Abnormal cardiac function	0 (0%)	0 (0%)	0(0%)

### Immune correlatives and transcriptome analysis results

**Figure 2:**
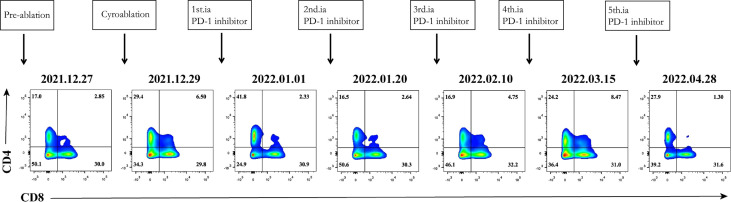
Immune correlative study of the combination therapy. Dynamic change of CD4+ T cells and CD8+ T cells.

**Figure 3:**
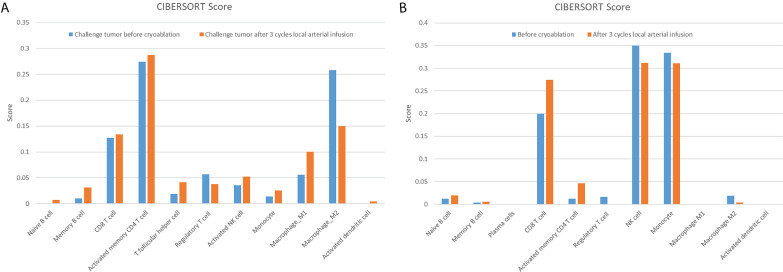
Challenged tumour **(A)** and matched peripheral blood **(B)** are associated with a variety of tumour-infiltrating immune cells. The related scores of eleven types of tumour-infiltrating immune cells, calculated using the CIBERSORT software, are shown in a bar plot.

Based on the results presented in **Figure 1**, changes in T-lymphocyte subsets during treatment were analysed using flow cytometry (**Figure 2**). The levels of CD4^+^ T cells increased to a peak on the second day after the combination therapy but significantly decreased before the second cycle of arterial perfusion, subsequently increasing in further cycles of treatment. To explore the relationship between tumour-infiltrating immune cells and the efficacy of combination therapy, biopsy specimens of the challenged tumour and matched peripheral blood samples collected from the patients before and after cryoablation were subjected to RNA-sequencing analysis, and tumour-infiltrating immune cells were further analysed using the CIBERSORT software. The levels of follicular T cells and antigen-presenting cells (macrophages and dendritic cells) increased after cryoablation and three cycles of arterial perfusion immunotherapy compared with those in the challenged tumour before treatment. The macrophage scores also showed an upward trend, with the levels of M2-type macrophages decreased and those of M1-type macrophages increased in the subcomponent classification, while regulatory T-cell levels decreased (**Figure 3A**). Peripheral blood analysis showed that the levels of regulatory T cells and M2 macrophages also decreased (**Figure 3B**).

## Discussion

Advanced solid cancers can use multiple adaptive immune resistance mechanisms to escape the attack of the immune system (20). Several studies have explored methods to improve the efficacy of immunotherapy to overcome tumour immune resistance (21–24); however, no clinical studies have demonstrated that cryoablation can overcome immune resistance. Given the disappointing prognostic outcome and limited treatment options in this cohort, how to choose the appropriate modalities after multiple lines of standard treatment, especially immunotherapy, needs to be studied urgently. Although the cohort size is small, however, some interesting and surprising efficacy which two out of nine (22.22%) patients achieved the best response (partial response) has also been found in the present study, and we believe that the disclosure, discussion, and sharing of the inspiring result through this paper are meaningful to some extent.

Sreya et al. (8) divided immune resistance into primary, acquired, and target-missing resistance. Primary resistance is intrinsic to tumour cells, and the most used biomarker to predict their response to ICIs is the expression level of PD-L1. Patients with melanoma and non-small cell lung cancer with low levels of PD-L1 expression had a response rate of only 10% when treated with PD-1 inhibitors, while patients with high PD-L1 expression had a response rate of 40–50% (25, 26). Another well-known biomarker for predicting an ICI response is the tumour mutation burden, which is strongly associated with the anti-PD-1 response in multiple cancer indications (27–29). The mechanism of acquired drug resistance is still unclear, and mutations in JAK1 and JAK2 in the interferon-γ response pathway may be the key to unveiling it. In a small cohort of patients with melanoma who exhibited acquired pembrolizumab resistance, the tumour outgrowth occurred in two patients harbouring loss-of-function mutations in these two proteins (30).

Cryoablation, known for its ability to destroy tumours by freezing them to lethal temperatures, has a potential secondary advantage in generating an antitumour immune response triggered by natural absorption of the malignant tissue. T-cell activation occurs when the unique T-cell receptor binds to the antigen produced upon cryoablation and is presented by antigen-presenting cells, such as macrophages and dendritic cells (31–33). In this study, the levels of CD4^+^ T cells increased while those of regulatory T cells decreased, compared with their pretreatment levels, when antigen-presenting cells (macrophages and dendritic cells) in the challenged tumour microenvironment were activated, which may have facilitated the process by increasing the levels of follicular T cells. Tumour-associated macrophages play an important role in the development of solid tumours (34). These macrophages can polarise into classically activated M1 or alternatively activated M2 states in response to different stimuli. M1 macrophages play an important role in tumour suppression by upregulating the expression of proinflammatory mediators and inducing an inflammatory response (35). In this study, the polarisation of macrophages to the M1 phenotype after cryoablation may have been related to the overcoming of immune resistance. These changes were also observed in circulating immune cells. However, the specific mechanisms involved require further study.

Circulating CD4^+^ T-cell levels showed a peak and subsequent decline during the combination therapy but continued to increase in subsequent therapy cycles; this may be associated with tumour remission, which is consistent with the challenged tumour shrinkage 10 weeks after cryotherapy, as described in another article (36), and may also be a PD-1 trailing effect (37).

Although cryoablation was effective in three patients with immune drug resistance in this study, there are still limitations worth discussing. First, the study had a small sample size, and further randomised multicentre studies are needed. Second, arterial infusion was used to deliver PD-1 in this study, but its effect has previously been mentioned only in the Shen’s study (38). It is worth evaluating whether changing the drug administration mode would be more beneficial for the efficacy of PD-1. Third, there is a question whether complete ablation of the tumour has an impact on overcoming immune resistance. It is well known that incomplete thermal ablation often promotes tumour progression (39–41). In terms of treatment choice, we preferred the lesions that could achieve complete ablation, but unfortunately, there are some that advocate not to destroy the tumour completely, to leave intact the TIL (Tumour Infiltrating Lymphocytes.) but unfortunately, some patients could not achieve complete ablation technically. limited by the sample size, our study could not confirm this correlation. Progressive disease found in all patients with incomplete cryoablation in this study requires further animal experiments for confirmation. Fourth, owing to the different types of cancer in the patients, the combination therapies were different in this study, including chemotherapy and tyrosine kinase inhibitors. Different combination therapies may have also affected the patient outcomes in this study. Prospective trials are needed to confirm whether cryoablation can overcome immune resistance.

## Conclusion

Cryoablation combined with arterial perfusion with programmed cell death protein 1 inhibitors has the potential to effectively and safely overcome immune resistance in patients with advanced solid cancers. The combination therapy leads to macrophage polarisation from the M2 to the M1 phenotype in the challenged tumour to enhance antitumour immunity.

## Data availability statement

The original contributions presented in the study are publicly available. This data can be found here: https://ngdc.cncb.ac.cn/gsa-human/s/5h1Es2P5.

## Ethics statement 

The studies involving human participants were reviewed and approved by the Ethics Committee of First Affiliated Hospital of Fujian Medical University. The patients/participants provided their written informed consent to participate in this study. Written informed consent was obtained from the individual(s) for the publication of any potentially identifiable images or data included in this article.

## Author contributions

ZL contributed to the study concept and design. FW, RG, YY, RL and JC contributed to the acquisition of clinical data. FW wrote the first draft of the manuscript. RG, YY, RL and JC supervised and oversaw the study. RL contributed to the statistical analysis. All authors contributed to the article and approved the submitted version.

## Conflict of interest

The research was conducted in the absence of any commercial or financial relationships that could be construed as a potential conflict of interest.

## Publisher’s note

All claims expressed in this article are solely those of the authors and do not necessarily represent those of their affiliated organizations, or those of the publisher, the editors and the reviewers. Any product that may be evaluated in this article, or claim that may be made by its manufacturer, is not guaranteed or endorsed by the publisher.
