# Supplementary material for: Investigation of the efficacy and safety of cryoablation and intra-arterial PD-1 inhibitor in patients with advanced disease not responding to checkpoint inhibitors: An exploratory study
Source: Front Immunol. 2022 Sep 23;13:990224. doi: 10.3389/fimmu.2022.990224 (PMC9537743; doi:10.3389/fimmu.2022.990224)
Supplement: Supplementary file 1 [file Table_1.docx]

**Supplementary table: Details of each patient**

|  | Age | Gender | Tumor type | TNM stage | Previous treatment | Cryoablated lesion | Cryoablation method | Artery selected | Cycles of artery infusion | Type of immune resistant | Best response |
| --- | --- | --- | --- | --- | --- | --- | --- | --- | --- | --- | --- |
| Patient 1 | 75 | male | Hepatocellular carcinoma | IV | TKI agent + PD-1 inhibitor | Chest wall metastases | Incomplete | External thoracic artery | 4 | acquired resistance | PD |
| Patient 2 | 58 | male | Adrenal carcinoma | IV | Operation/chemotherapy/radiotherapy/TKI agent + PD-1 inhibitor | Chest wall metastases | Incomplete | External thoracic artery | 3 | primary resistance | PD |
| Patient 3 | 44 | male | Hepatocellular carcinoma | IV | Operation/ablation/TKI agent + TACE/TKI + PD-1 inhibitor | Pelvic metastases | Complete | [Internal iliac artery](javascript:;) | 4 | acquired resistance | SD |
| Patient 4 | 64 | male | Hepatocellular carcinoma | IIIA | D-TACE/radiotherapy/TKI agent+ PD-1 inhibitor | Liver cancer | Incomplete | Hepatic artery | 3 | primary resistance | PD |
| Patient 5 | 66 | female | Gallbladder carcinoma | IV | Operation/chemotherapy/chemotherapy + PD-1 inhibitor/TKI + PD-1 inhibitor | Abdominal metastases | Incomplete | Gastroduodenal artery | 4 | primary resistance | PD |
| Patient 6 | 69 | male | Hepatocellular carcinoma | IV | Operation/TKI agent/TKI agent + PD-1 inhibitor | Liver cancer | Complete | Hepatic artery | 4 | primary resistance | PD |
| Patient 7 | 38 | male | Hepatocellular carcinoma | IV | Operation/TKI agent +TACE+ radiotherapy /TKI agent + PD-1 inhibitor | Liver cancer | Complete | Hepatic artery | 6 | acquired resistance | PD |
| Patient 8 | 43 | male | Pancreatic cancer | IV | Chemotherapy/radiotherapy/ chemotherapy + TKI + PD-1 inhibitor | [Liver metastases](javascript:;) | Complete | Hepatic artery | 5 | acquired resistance | PR |
| Patient 9 | 45 | male | Hepatocellular carcinoma | IV | TACE/operation/TKI agent/TKI agent + PD-1 inhibitor | Pulmonary metastases | Complete | Bronchial artery | 6 | acquired resistance | PR |

Abbreviations: TACE, Transhepatic Arterial Chem Otherapy And Embolization; TKI, Tyrosine Kinase Inhibitor; PR, partial response; SD, stable disease; PD, progressive disease.
